# Hypocomplementemia dynamics during tocilizumab therapy in systemic juvenile idiopathic arthritis: a retrospective longitudinal study

**DOI:** 10.3389/fimmu.2025.1607101

**Published:** 2025-08-29

**Authors:** Jiayun Ling, Qingfang Zhou, Fang Xie, Xiaohui Liu

**Affiliations:** Department of Rheumatology and Immunology, Jiangxi Children’s Hospital, Nanchang Medical College, Nanchang, Jiangxi, China

**Keywords:** systemic juvenile idiopathic arthritis, tocilizumab, hypocomplementemia, disease activity, infection

## Abstract

**Objective:**

This study aimed to investigate the trajectory of complement C3 and C4 levels during tocilizumab (TCZ) treatment in patients with systemic juvenile idiopathic arthritis (sJIA), explore the dynamic relationship between hypocomplementemia and disease activity, and characterize adverse events during long-term TCZ therapy.

**Methods:**

A retrospective analysis was conducted on 19 sJIA patients diagnosed according to the 2019 PRINTO criteria. Clinical data, including C3 and C4 levels, disease activity (sJADAS10-ESR score), and adverse events, were collected at baseline and at intervals from 2 to 96 weeks following TCZ initiation. Statistical analyses were conducted accordingly.

**Results:**

The 19 analyzed patients (6 males, 31.58%; 13 females, 68.42%) had a median (IQR) age of disease onset of 6.75 (4.58-9.42) years and a median (IQR) follow-up duration of 2.08 (1.83-2.67) years. After 2 weeks of TCZ treatment, median (IQR) serum C3 levels declined from 1.45(1.27-1.75) g/L at baseline to 1.11 (0.97-1.18) g/L (a 23.45% reduction, *P =* 0.009), and C4 levels decreased from 0.29 (0.21-0.38) g/L to 0.13 (0.09-0.17) g/L (a 55.17% reduction, *P* = 0.005). At week 48, hypocomplementemia was observed in 68.42% of patients for C3 and 26.32% for C4. The mixed linear model revealed significant reductions in C3 (*β* = -0.058, *P <* 0.001), C4 (*β* = -0.061, *P* = 0.022), and sJADAS10-ESR scores (*β* = -0.628, *P <* 0.001) across all time points compared to baseline. Longitudinal Spearman analysis revealed a positive correlation between complement levels and disease activity at specific stages: C3 (r = 0.529, *P* = 0.029) and C4 (r = 0.577, *P* = 0.015) were most strongly correlated with sJADAS10-ESR at week 24. Notably, C3 remained significantly correlated at week 48 (r = 0.513, *P* = 0.025). Acute upper respiratory tract infections were the most common adverse events (occurring in 63.16% of patients), while no serious infections or new autoimmune diseases were reported.

**Conclusions:**

Complement C3 and C4 levels during TCZ treatment follow a trajectory characterized by a rapid early decline followed by a sustained low-level plateau. Long-term hypocomplementemia appears to be well tolerated, with no increased risk of serious infections or autoimmune complications. These findings suggest that hypocomplementemia reflects deep IL-6 signaling inhibition rather than pathological complement consumption.

## 1 Introduction

Systemic juvenile idiopathic arthritis (sJIA) is a pediatric rheumatic disease characterized by systemic inflammation and immune dysregulation (1). Interleukin-6 (IL-6) plays a pivotal role in its pathogenesis by binding to a receptor complex composed of the IL-6 receptor (IL-6R) and two gp130 molecules (2, 3). This interaction activates the Janus kinase (JAK)-signal transducer and activator of transcription (STAT) pathway, which upregulates pro-inflammatory gene expression and drives disease progression (4). Tocilizumab (TCZ), a humanized monoclonal antibody targeting IL-6R, has become a cornerstone therapy for sJIA. By blocking IL-6 signaling, TCZ effectively reduces systemic inflammation and joint symptoms (5, 6). Consequently, it is now recommended as a first-line biologic agent in international treatment guidelines (7).

The complement system, a key component of innate immunity, contributes to pathogen clearance and regulation of inflammatory responses (8). Complement components C3 and C4, primarily synthesized in the liver, are positively regulated by IL-6 (9). In both rheumatoid arthritis (RA) and juvenile idiopathic arthritis (JIA), C3 and C4 play essential roles in disease pathogenesis. Genetic variations, copy number alterations, and autoantibodies targeting these components have been linked to disease susceptibility, severity, and clinical phenotype (10–13). In RA, complement activation is closely associated with disease progression, with synovial deposition of complement components contributing to exacerbated inflammation and joint damage (14, 15). Recent clinical observations suggest that TCZ treatment induces a persistent decline in serum C3 and C4 levels across various rheumatic diseases (16, 17). However, the temporal dynamics of complement reduction, its relationship with disease activity, and the long-term clinical implications in sJIA remain unclear. To investigate these unclear points, a 96-week longitudinal study was conducted to assess changes in complement C3 and C4 levels during TCZ therapy in sJIA. The investigation focused on (1) the temporal patterns of hypocomplementemia, (2) its correlation with disease activity, and (3) the long-term implications of TCZ-induced complement reduction.

## 2 Methods

### 2.1 Patients

This single-center retrospective cohort study included patients newly diagnosed with sJIA who received sequential TCZ treatment at the Department of Rheumatology and Immunology, Jiangxi Children’s Hospital, between January 2021 and May 2024. The study protocol was approved by the Ethics Committee of Jiangxi Children’s Hospital (Approval No: JXSEYY-YXKY-20240174).

### 2.2 Inclusion and exclusion criteria for study subjects

#### 2.2.1 Inclusion criteria

Patients meeting the following criteria were included: ① Diagnosis of sJIA based on the 2019 criteria established by the Pediatric Rheumatology International Trials Organization (PRINTO) (18). ② Aged ≤ 18 years at the time of diagnosis. ③Received standard TCZ treatment (12 mg/kg for body weight ≤30kg and 8 mg/kg for body weight > 30kg). After 12 weeks, the dosing interval could be extended according to clinical remission status. All patients received at least 12 TCZ infusions, and the treatment duration was ≥48 weeks. ④ Did not receive other biologic agents or drugs known to modulate complement levels during the treatment period.

#### 2.2.2 Exclusion criteria

Patients were excluded if they met any of the following criteria: ①Coexistence of primary conditions known to affect complement levels, including systemic lupus erythematosus, hereditary complement deficiencies, or nephrotic syndrome. ② Baseline hypocomplementemia (C3 < 0.90 g/L or C4 < 0.10 g/L). ③ Development of secondary complement-consuming conditions during treatment, such as sepsis, streptococcal infections, or severe liver failure. ④ Incomplete clinical records, including missing disease activity scores or treatment data, or loss to follow-up.

### 2.3 Collection and observation indicators

#### 2.3.1 Baseline data

The following baseline characteristics were collected: demographics (age, gender), disease activity (sJADAS10-ESR score), concurrent medication use (glucocorticoid dosage and methotrexate administration), autoantibody profiles, and hepatic and renal function parameters.

#### 2.3.2 Core observation indicators

Dynamic monitoring of complement levels: Serum C3 and C4 levels were measured at baseline and at 2, 4, 8, 24, 48, and 96 weeks after initiation of TCZ treatment. Measurements were performed using immunoturbidimetric assays. Normal reference ranges were defined as follows: C3, 0.90–1.80 g/L; C4, 0.10–0.40 g/L. Hypocomplementemia was defined as C3 < 0.90 g/L and/or C4 < 0.10 g/L.

Disease activity assessment: Disease activity was evaluated using sJADAS10-ESR, which incorporates erythrocyte sedimentation rate (ESR), body temperature, active joint count, and global assessments by both physicians and parents.

Adverse events: Adverse events, including infections, new-onset autoimmune diseases, and malignancies, were recorded during the treatment period. Severe infections were defined as those posing a life-threatening risk or leading to severe complications.

### 2.4 Statistical analysis

All statistical analyses were conducted using SPSS Statistics version 26.0 (IBM, USA). Continuous variables were expressed as medians with interquartile ranges (IQR), and categorical variables were presented as frequencies and percentages. Data distribution was assessed using the Kolmogorov–Smirnov test in combination with histogram inspection. Results indicated that serum C3 and C4 levels followed a gamma distribution, while sJADAS10-ESR scores conformed to a Poisson distribution. Changes in complement levels (C3 and C4) and sJADAS10-ESR scores before and after TCZ treatment were analyzed using the Wilcoxon signed-rank test. Longitudinal changes in complement levels were assessed using a generalized linear mixed model (gamma regression with log link function), and disease activity was analyzed using a generalized linear mixed model with a Poisson distribution and log link function. Spearman rank correlation analysis was employed to evaluate associations between serum C3/C4 levels and sJADAS10-ESR scores at each post-treatment time point. Adverse events were summarized using cumulative frequencies and the proportion of patients affected. Statistical significance was defined as a two-sided *P*-value < 0.05.

## 3 Results

### 3.1 Clinical characteristics and follow-up

According to the inclusion criteria, 20 patients were initially enrolled in the study, with 1 excluded due to incomplete clinical records. As summarized in **Table 1**, this retrospective study included 19 patients (6 males, 31.58%; 13 females, 68.42%) with a median (IQR) age of disease onset of 6.75 (4.58-9.42) years. The median (IQR) follow-up duration was 2.08 (1.83-2.67) years. Before TCZ treatment, 94.74% (18/19) of patients were treated with glucocorticoids, and 73.68% (14/19) were treated with methotrexate. The median (IQR) sJADAS10-ESR score was 31.30 (25.20-35.00), indicating high disease activity in all cases. After 48 weeks of TCZ therapy, all patients discontinued glucocorticoid treatment, 36.84% (7/19) discontinued methotrexate treatment, and the sJADAS10-ESR score decreased to 0 (0-5.00).

**Table 1:** Baseline characteristics of systemic juvenile idiopathic arthritis patients treated with tocilizumab (n=19).

Patient number	Sex	Age at onset (years)	Pre-TCZ treatment					Post- TCZ treatment (48 Weeks)					Minimum C3 level (g/L)	Timepoint (weeks)	Minimum C4 level (g/L)	Time point (weeks)	Follow-up (years)	Comorbid autoimmune disease
			sJADAS-ESR score	GC (Yes/No)	MTX (Yes/No)	C3 (g/L)	C4 (g/L)	sJADAS-ESR score	GC (Yes/No)	MTX (Yes/No)	C3 (g/L)	C4 (g/L)						
1	Male	4.92	34.80	Yes	Yes	2.10	0.43	10.00	No	No	1.13	0.40	0.61	78	0.07	78	3.42	No
2	Female	6.75	31.40	Yes	Yes	2.33	0.38	0.50	No	Yes	0.70	0.09	0.70	52	0.09	52	3.25	No
3	Female	4.67	39.00	Yes	Yes	1.37	0.10	2.00	No	Yes	0.63	0.01	0.55	104	0.01	52	2.67	No
4	Female	2.67	35.50	Yes	Yes	1.62	0.21	0	No	Yes	0.84	0.11	0.55	78	0.01	20	2.08	No
5	Male	5	25.00	Yes	Yes	1.75	0.35	3.50	No	Yes	0.82	0.12	0.45	104	0.01	104	3.42	No
6	Female	9.42	35.00	Yes	Yes	1.66	0.2	0.50	No	Yes	0.95	0.11	0.55	16	0.06	16	2.33	No
7	Female	9.17	25.20	Yes	Yes	1.75	0.29	0	No	Yes	0.81	0.10	0.55	30	0.04	8	2.50	No
8	Male	6.67	34.10	Yes	Yes	0.96	0.58	0	No	Yes	0.76	0.10	0.69	38	0.06	26	1.67	No
9	Female	4.58	30.10	Yes	Yes	1.84	0.38	9.00	No	Yes	1.92	0.49	0.58	34	0.08	30	1.50	No
10	Female	14.25	21.60	Yes	Yes	1.12	0.13	5.00	No	Yes	0.82	0.09	0.71	38	0.01	26	1.83	No
11	Male	3.92	19.00	Yes	Yes	1.22	0.35	0	No	No	0.81	0.14	0.66	56	0.08	8	2.08	No
12	Male	7.25	10.60	Yes	Yes	1.27	0.24	0	No	Yes	0.90	0.28	0.74	52	0.11	52	2.92	No
13	Female	7.83	31.30	Yes	No	1.70	0.45	1.50	No	No	0.80	0.13	0.60	16	0.09	16	1.83	No
14	Female	7.08	19.00	No	No	1.45	0.29	0	No	No	0.71	0.11	0.54	34	0.08	30	1.83	No
15	Female	10.33	37.70	Yes	No	1.42	0.37	1.00	No	No	1.33	0.29	0.77	82	0.09	2	2.58	No
16	Male	14.92	32.70	Yes	No	1.63	0.57	7.00	No	No	1.13	0.29	0.93	64	0.20	64	2.50	No
17	Female	1.33	30.20	Yes	No	1.38	0.21	0	No	No	0.73	0.09	0.68	16	0.07	16	1.83	No
18	Female	12.33	28.50	Yes	Yes	1.11	0.24	0	No	Yes	0.74	0.10	0.58	68	0.04	8	1.91	No
19	Female	3.83	38.00	Yes	Yes	1.28	0.28	0	No	Yes	0.64	0.09	0.64	42	0.09	42	1.08	No

GC, Glucocorticoid; MTX, Methotrexate; ANA, Antinuclear antibody; U1RNP, U1 small nuclear ribonucleoprotein; Sm, Smith antigen; SSA, Sjögren's syndrome-related antigen A; Ro-52, Ro ribonucleoprotein 52kDa.

### 3.2 Complement level changes and correlation with disease activity

**Figure 1:**
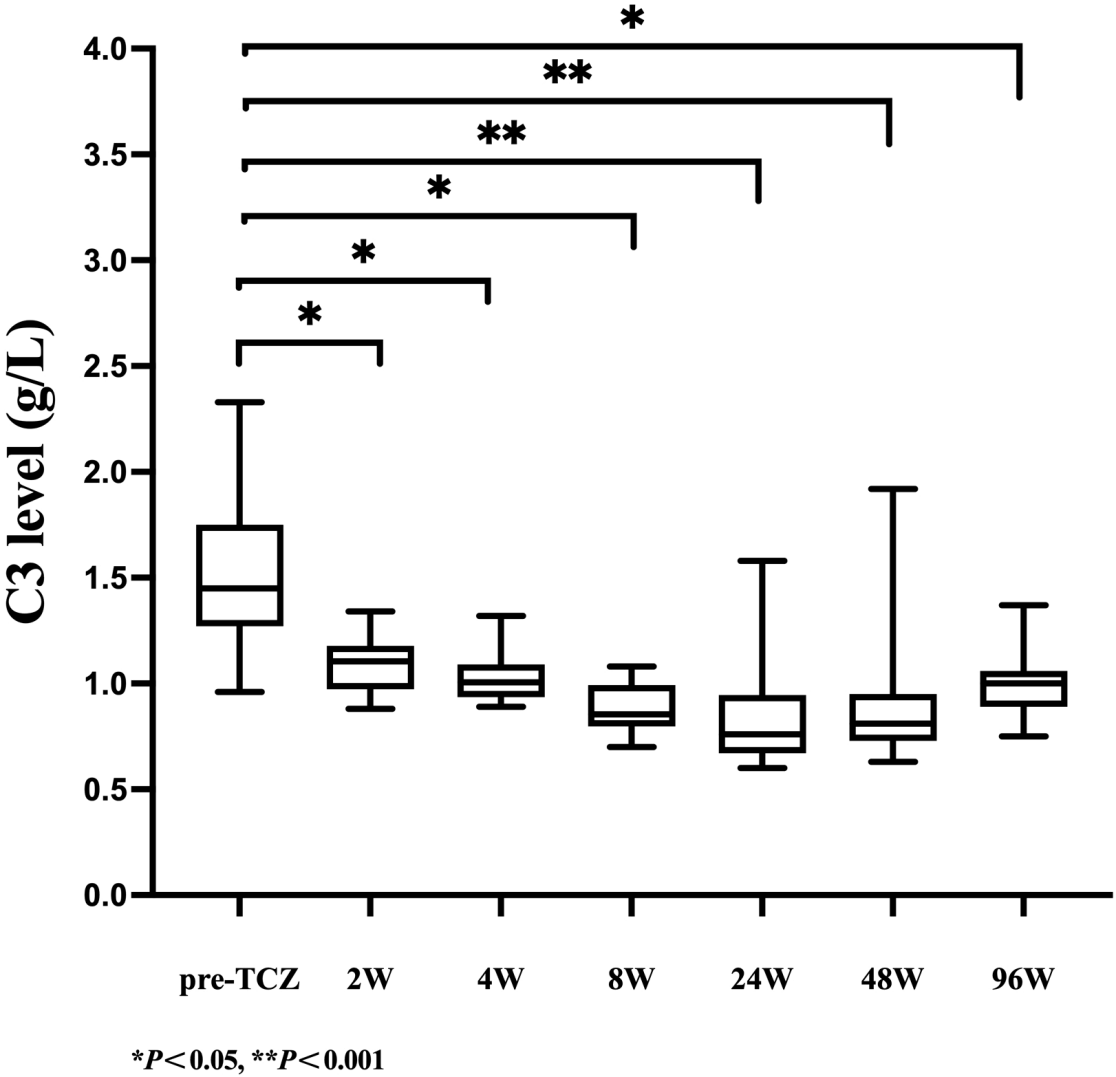
Longitudinal changes in serum C3 levels during TCZ treatment.

**Figure 2:**
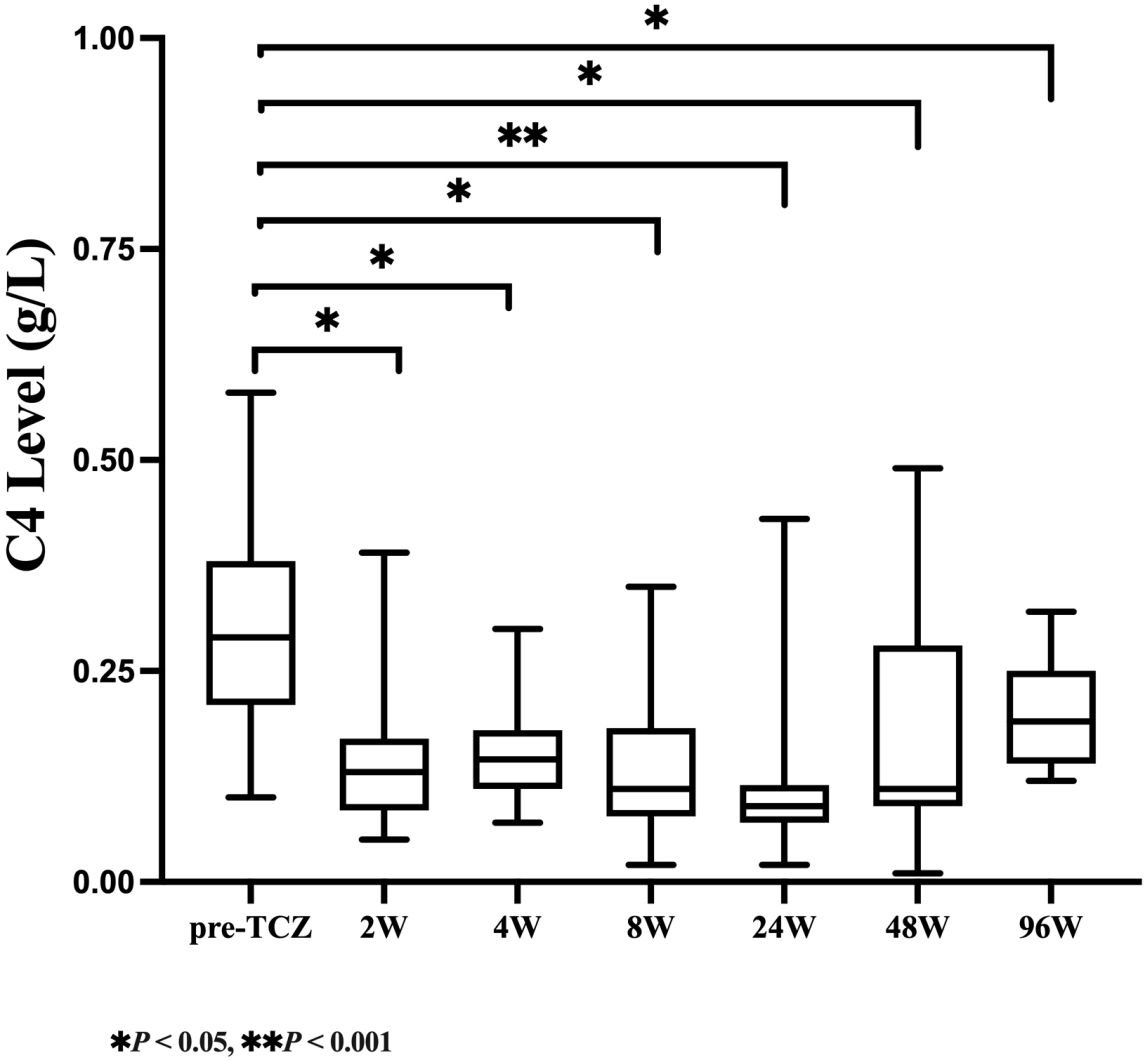
Longitudinal changes in serum C4 levels during TCZ treatment.

As shown in **Table 2**, serum C3 levels declined rapidly after 2 weeks of TCZ treatment, with the median (IQR) value decreasing from 1.45 (1.27-1.75) g/L at baseline to 1.11 (0.97-1.18) g/L (a 23.45% reduction, *P*=0.009). Over the same period, a more pronounced decrease was observed in C4 levels, which dropped from 0.29 (0.21-0.38) g/L to 0.13 (0.09-0.17) g/L (a 55.17% reduction, *P* = 0.005. At week 48, the median (IQR) C3 level remained at 0.81 (0.73-0.95) g/L, with 68.42% of patients developing C3 hypocomplementemia. The median (IQR) C4 level was 0.11 (0.09-0.28) g/L, and 26.32% of patients exhibited C4 hypocomplementemia. By week 96, both C3 and C4 levels had slightly recovered to 1.00 (0.89-1.06) g/L and 0.19 (0.14-0.25) g/L, respectively, but remained significantly lower than baseline (with reductions of 31.03% and 34.48%, respectively, *P* < 0.05). As illustrated in **Figures 1**, **2**, complement levels followed a trajectory characterized by a rapid early decline and a sustained low-level plateau, without evidence of rebound over time or in response to extended TCZ dosing intervals.

**Table 2:** Longitudinal changes in serum C3/C4 levels and disease activity during tocilizumab treatment.

Time point	C3 (g/L)	P*	C4 (g/L)	P*	sJADAS10-ESR score	P*
pre-TCZ	1.45 (1.27-1.75)**	–	0.29 (0.21-0.38)	–	31.30 (25.20-35.00)	–
2 Weeks	1.11 (0.97-1.18)	0.009	0.13 (0.09-0.17)	0.005	9.00 (5.00-10.50)	<0.001
4 Weeks	1.00 (0.94-1.09)	0.003	0.15 (0.11-0.18)	0.002	5.50 (3.00-7.50)	<0.001
8 Weeks	0.86 (0.80-0.99)	0.001	0.11 (0.08-0.18)	0.001	2.00 (1.50-4.50)	<0.001
24 Weeks	0.76 (0.67-0.95)	<0.001	0.09 (0.07-0.12)	<0.001	1.00 (1.00-4.50)	<0.001
48 Weeks	0.81 (0.73-0.95)	*<*0.001	0.11 (0.09-0.28)	0.001	0 (0-5.00)	<0.001
96 Weeks	1.00 (0.89-1.06)	0.001	0.19 (0.14-0.25)	0.006	0 (0-5.50)	0.001

*P-values from Wilcoxon signed-rank tests versus baseline (pre-TCZ).

**Values expressed as median (interquartile range).

The mixed linear model (**Table 3**) confirmed significant downward trends over time in C3 (*β* = - 0.058, *P* < 0.001), C4 (*β* = -0.061, *P =* 0.022), and sJADAS10-ESR (*β* = -0.628, *P* < 0.001). Longitudinal Spearman correlation analysis revealed stage-specific associations. As shown in **Table 4**, C3 levels were positively correlated with *sJADAS10* - *ESR* at both week 24 (r = 0.529, *P* = 0.029) and week 48 (r = 0.513, *P* = 0.025). As shown in **Table 5**, a significant correlation between C4 and disease activity was observed only at week 24 (r = 0.577, *P* = 0.015). However, no significant correlations were observed at baseline or at weeks 2, 4, 8, or 96(all *P* > 0.05).

**Table 3:** Mixed linear models comparing C3- C4, and sJADAS10-ESR levels across treatment time points relative to baseline.

Parameter	Coefficient (*β*)	*P*-value	95% Confidence interval
C3 (g/L)	-0.058	<0.001	(-0.082, -0.035)
C4 (g/L)	-0.061	0.022	(-0.112, -0.009)
sJADAS10-ESR Score	-0.628	<0.001	(-0.796, -0.461)

**Table 4:** Spearman correlation between serum C3 and disease activity (sJADAS10-ESR) during tocilizumab therapy.

Time point	pre-TCZ		2 Weeks		4 Weeks		8 Weeks		24 Weeks		48 Weeks		96 Weeks	
sJADAS10 - ESR Score	*r*	*P*	*r*	*P*	*r*	*P*	*r*	*P*	*r*	*P*	*r*	*P*	*r*	*P*
	0.119	0.626	0.006	0.986	-0.004	0.991	-0.123	0.675	0.529	0.029	0.513	0.025	0.103	0.715

**Table 5:** Spearman correlation between serum C4 and disease activity (sJADAS10-ESR) during tocilizumab therapy.

Time point	pre-TCZ		2 Weeks		4 Weeks		8 Weeks		24 Weeks		48 Weeks		96 Weeks	
sJADAS10 - ESR Score	*r*	*P*	*r*	*P*	*r*	*P*	*r*	*P*	*r*	*P*	*r*	*P*	*r*	*P*
	0.005	0.983	-0.056	0.877	-0.080	0.806	-0.070	0.812	0.577	0.015	0.400	0.090	-0.191	0.496

### 3.3 Adverse events

A total of 62 adverse events were documented during a median (IQR) follow-up period of 2.08 (1.83-2.67) years, as detailed in **Table 6**. Infectious events were primarily composed of acute upper respiratory tract infections, reported in 63.16% of patients (12/19), followed by acute bronchitis in 42.11% (8/19) and pneumonia in 10.53% (2/19). Among non-infectious events, the most frequent were abnormal liver function in 21.05% (4/19) and leukopenia in 15.80% (3/19). No cases of severe infection, new-onset autoimmune disease, or malignancy were observed during the follow-up period. One patient (5.26%) developed a duodenal bulb ulcer with gastrointestinal bleeding, which was attributed to the combined use of glucocorticoids and non-steroidal anti-inflammatory drugs.

**Table 6:** Adverse events during tocilizumab treatment in systemic juvenile idiopathic arthritis (n=19).

Adverse events*	Total events n (%)	Affected patients n (%)
Acute Upper Respiratory Tract Infection	31 (50.00)	12 (63.16)
Acute Bronchitis	13 (20.97)	8 (42.11 )
Leukopenia	5 (8.06)	3 (15.80 )
Abnormal Liver Function	4 (6.45)	4 (21.05)
Pneumonia	3 (4.84)	2 (10.53)
Papular Urticaria	2 (3.23)	1 (5.26)
Oral Candidiasis	1 (1.61)	1 (5.26 )
Ulcerative Stomatitis	1 (1.61)	1 (5.26 )
Infectious Mononucleosis	1 (1.61)	1 (5.26 )
Duodenal bulbar ulcer with bleeding	1(1.61)	1 (5.26 )

*Median (interquartile range) follow-up time: 2.08 (1.83, 2.67) years.

## 4 Discussion

This study is the first to systematically characterize the dynamic trajectory of complement C3 and C4 levels during TCZ treatment in sJIA patients. The results identified a distinct pattern: a rapid early decline in complement levels followed by a prolonged plateau at low levels. These findings offer important longitudinal insights into the impact of TCZ on the complement system and add valuable details to the literature regarding long-term complement dynamics in sJIA.

The observed trends are consistent with findings from previous studies involving other autoimmune diseases. In 2018, Romano et al. conducted a small-scale investigation in RA patients treated with TCZ, reporting significant reductions in serum C3 and C4 levels, particularly in C4, as early as four weeks after treatment initiation. The proportion of patients with complement levels below the normal range increased with treatment duration (16). Similarly, Bieber et al. retrospectively analyzed 108 patients with various autoimmune conditions, including 8 sJIA patients, and found that 30% experienced C4 reductions and 21% experienced C3 reductions following TCZ therapy (17). In patients with Takayasu arteritis, TCZ treatment was also associated with significant post-treatment declines in C3 and C4 levels, correlating with improvements in inflammatory markers and disease activity (19). Furthermore, Iván Ferraz-Amaro et al. performed serial functional complement assays in 27 RA patients, demonstrating decreased activity in both the classical and alternative pathways after TCZ treatment, while the lectin pathway remained unaffected (20). Collectively, these findings indicate that TCZ-associated reductions in complement levels represent a common biological response across multiple autoimmune diseases. The observed complement-lowering effect appears to be mechanistically distinct from those of other commonly used treatments, such as glucocorticoids or JAK inhibitors. The findings suggest that TCZ-induced hypocomplementemia likely reflects deep suppression of IL-6 signaling, which may exert unique immunomodulatory effects not shared by other agents.

The reduction in complement C3 and C4 levels observed during TCZ treatment is likely closely associated with inhibition of the IL-6 signaling pathway. IL-6 plays a pivotal role in stimulating hepatic synthesis of acute-phase proteins, including C3 and C4 (21, 22). A study on COVID-19 and complement regulation demonstrated enhanced activation of the complement system in infected patients, particularly through the alternative pathway. IL-6 was identified as a key upstream regulator, and treatment targeting IL-6 resulted in decreased levels of complement activity and related components (23). Similarly, data from clinical trials investigating TCZ in patients with systemic lupus erythematosus (SLE) have shown continued reductions in C3 and C4 levels despite clinical improvement. These trials also reported significant declines in complement activation products, such as iC3b and C5b-9, suggesting that the reductions reflect suppressed synthesis rather than pathological consumption (24–26). The complement system interacts with innate immune sensors, including Toll-like receptors (TLRs). In particular, TLR4 activation can upregulate IL-6 family cytokines, further amplifying inflammation (27). In sJIA, systemic inflammation may enhance IL-6 production through TLRs, complement receptors, and other innate immune alarm receptors (28). Inhibiting IL-6 signaling with TCZ may disrupt this pro-inflammatory equilibrium, thereby influencing complement synthesis. To date, no clinical studies have specifically reported hypocomplementemia following the use of other IL-6 receptor antagonists in rheumatic diseases. However, recent findings involving sarilumab, another IL-6 receptor blocker, demonstrated that its administration attenuated chondrocyte senescence induced by the terminal complement complex (TCC) following trauma or exposure to human serum. These data suggest that IL-6 may be involved in TCC-mediated stress-induced premature senescence (29).

In this study, progressive TCZ treatment was accompanied by a gradual decline in sJADAS10-ESR scores, indicating reduced disease activity, alongside continuous reductions in C3 and C4 levels. At weeks 24 and 48, a significant positive correlation was observed between C3 levels and sJADAS10-ESR scores, suggesting a potential link between complement dynamics and disease activity in sJIA. These findings are consistent with previous research. E. Conticini et al. demonstrated that C3 and C4 levels were associated with disease activity in patients with giant cell arteritis. In the TCZ-treated group, reductions in C3 (*P* =0.001) and C4 (*P <*0.001) were more pronounced and occurred independently of CRP or ESR levels, indicating that C4 might serve as a potential biomarker for monitoring disease activity and therapeutic response (30). However, conflicting evidence exists. Iván Ferraz-Amaro et al. found no significant correlation between changes in disease activity and functional alterations in the three complement pathways among RA patients treated with TCZ. Additionally, complement activation appears to vary across different JIA subtypes (20). Hackl et al. analyzed 60 JIA patients (including 7 sJIA patients), and found that terminal complement complex (TCC) levels were significantly elevated during the acute phase in certain subtypes (e.g., polyarticular JIA and extended oligoarticular JIA). However, sJIA patients showed no complement activation in TCC or COMPL300 analyses, suggesting a distinct immunological profile (31). The hypocomplementemia observed during TCZ treatment is likely due to IL-6-mediated suppression of complement synthesis rather than complement depletion through immune activation. Consequently, the decline in C3 and C4 levels during treatment may partially reflect clinical improvement. However, current evidence remains insufficient to support their use as standalone biomarkers for continuous monitoring of disease activity in sJIA.

Throughout TCZ treatment, adverse events including infections, leukopenia, and abnormal liver function were documented in our patients. However, the incidence of these events was not significantly higher than that reported in previous studies (26, 32, 33). Early case reports described an RA patient developing new-onset glomerulonephritis with positive dsDNA and low complement levels following TCZ treatment (34). However, Romano et al. examined 19 patients treated with TCZ and found no clinical signs of glomerulonephritis or increased incidence of immune complex–related complications, such as recurrent fever (16). Similarly, Illei et al. found no correlation between complement levels and infection risk in TCZ-treated lupus patients (24). Furthermore, Bieber et al. also found that low complement levels during treatment were not associated with an increased incidence of infections, secondary immunodeficiency, or malignancies (17). Although long-term hypocomplementemia was frequently observed in the present study, no significant increase in infection risk was detected. One possible explanation is that TCZ suppresses the classical complement pathway while preserving lectin pathway activity, which may provide partial compensation for innate immune function. Nonetheless, rare adverse events warrant continued vigilance. Given that the median follow-up duration was 2.08 years, extended observation periods of five years or longer are needed to exclude delayed complications such as malignancies or autoimmune sequelae. Given the critical role of the complement system in immune surveillance, patients with persistently reduced complement levels during prolonged TCZ therapy should undergo close monitoring, particularly for infectious complications.

The study demonstrated that TCZ-induced hypocomplementemia serves as a biomarker of profound IL-6 pathway inhibition, rather than a direct indicator of pathological complement consumption or disease activity. Although certain adverse events occurred during TCZ therapy, no significant increase in infection risk was observed, supporting the continued use of TCZ for disease management under hypocomplementemic conditions. However, due to methodological limitations, including a single-center retrospective design and small sample size, serum C3 and C4 levels cannot be regarded as reliable longitudinal markers for monitoring disease activity in sJIA. Future investigations should prioritize multicenter, large-scale prospective studies to clarify the compensatory mechanisms of the complement system. In addition, further exploration of the relationship between dynamic changes in complement fragments (such as C3a and C5b-9) and organ-specific injury may offer deeper insight into complement alterations during TCZ treatment and their clinical relevance in sJIA.

## Data Availability

The original contributions presented in the study are included in the article/**Supplementary Material**. Further inquiries can be directed to the corresponding authors.
